# Book Reviews

**Published:** 1902-12

**Authors:** 


### BOOK REVIEWS.

The Principles and Practice of Bandaging. By Gwilym G. Davis, M. D., Assistant Professor of Applied Anatomy, University of Pennsylvania. 12mo, pp. 157. Illustrated from Original Drawings by the Author. Philadelphia: P. Blakiston's Son & Company. 1902. [Price, $1.50 net.]

This small treatise has been greatly improved since the first edition was issued, now about eleven years ago. The text has been rewritten and the illustrations, 163 in all, have been redrawn, hence it is practically a new book. The classic roller bandage is given its appropriate place of importance, detailed directions being given for its construction and application. This is important, for skill in its application once thoroughly acquired is never forgotten, and it furnishes the foundation for all other bandages and bandaging.

The introduction of gauze for the construction of bandages has somewhat lessened the importance of instruction in the art of bandaging, because many suppose that it will do the work no matter how indifferently or carelessly applied. This work will do much to remove this erroneous impression from the minds of those who have made this mistake. The text is clear and the illustrations are excellent. The book is intended for the beginner, but it will do the practitioner no harm to freshen his knowledge of the subject by a careful examination of its pages.

A Physician's Practical Gynecology. By W. O. Henry, M. D., Professor of Gynecology in the Creighton Medical College, Omaha, Neb. First edition. 226 pages, 5 full-page illustrations and 61 others in the text. Lincoln, Neb.. The Review Press. 1902. [Price, cloth, $2.00.]

The attempt to present the elementary principles of gynecology in a fashion that will appeal to the understanding of students has been made once and again with failures; now and then success on these lines has been obtained, but it is not the rule. In the present instance the author has pretty nearly hit the mark. The first point in this book that impresses us favorably is that the pronunciation of "gynecology" is given correctly. Since the word came into general use the sins against orthoepy have been multiplied an unspeakable number of times. The old-fashioned "guy-necologist," however, is approaching with more or less speed his appropriate place in the museum of orthopeic history. It is remarkable that any such abomination against the laws of good pronunciation ever could have been practised by educated men. Jĭn-ē-kol'-o-jĭ, the pronunciation given in this book, is the *only* way the word should be pronounced, as a distinguished medical philologist remarked to us several years ago, and Henry will have done medicine a good service if he succeeds in correcting this error of speech among the young men in the west.

The general arrangement of the manual is good and its material is well chosen for the undergraduate and general practitioner of medicine, for whom it is particularly designed. The paper and letter press are excellent and the make-up of the book is appropriate.

---

Gibson and Russell's Physical Diagnosis. Third edition, revised and rewritten by Francis D. Boyd, C. M. G., M. D., F. R. C. P. E., Assistant Physician, Edinburgh Royal Infirmary. 12mo, pp. 465. With 144 illustrations. New York: D. Appleton & Company. Edinburgh and London: Young J. Pentland. 1902.

Many changes of methods and some advances have been made since the first edition of this book, which is in reality a manual, was issued, now some twelve years ago. This has made necessary not only revision but rewriting a considerable part of the text. The new sections added deal with examination of the blood, examination of the gastric contents, intestinal parasites, the cranial nerves, and clinical bacteriology. Revision has been made of the section on sense of sight, by Dr. George Mackay, who originally prepared it, and whose skill in this department is widely known. Other special sections have been properly revised by the several gentlemen who were responsible in whole or in part for their original preparation.

The illustrations have been largely increased in this edition, but these have not kept pace with the text in improvement. Taken in its entirety, however, the volume is a safe and useful guide to physical diagnosis. It is especially commended to the student and junior practitioner for its practical character, accuracy of teaching, and compact form. Dr. Boyd deserves special praise for the conscientious manner in which he has met the task that has fallen to him as the responsible editor of the third edition of this excellent manual.

---

Diseases of the Stomach. Their Special Pathology, Diagnosis and Treatment, with sections on Anatomy, Physiology, Chemical and Microscopical Examination of Stomach Contents, Dietetics, Surgery of the Stomach, etc. By John C. Hemmeter, M. D., Philos D., Professor in the Medical Department of the University of Maryland, Baltimore. Third revised and enlarged edition. Octavo, pp. 883. Illustrated. Philadelphia: P. Blakiston's Son & Co. 1902. [ Price, $6.00 net.]

The author of this treatise has abundant reason for congratulation, in the conspicuous eminence the previous two editions have given him as a student of the subject he has chosen for special research, and as a clear writer upon it. His teachings as set forth in these books have made him famous the world over.

The columns of the Journal heretofore have stated in unmistakable language our opinion of this work in the notices of the first and second editions. In May, 1898, we took up the treatise in considerable detail, discussing it section by section. In dealing with the second edition in August, 1900, we were but little less extended in our consideration of the work. We beg to refer our readers to those notices as being equally appropriate today in their application. The science it is true has progressed, but so has Hemmeter, and this new edition has given him the opportunity to add such new material as seemed necessary to keep his treatise in the forefront of professional knowledge.

It is quite certain that this treatise will continue to be, as heretofore, not second to any other textbook on diseases of the stomach.

---

The Medical Student's Manual of Chemistry. By R. A. Witthaus, A. M., M. D., Professor of Chemistry, Physics and Toxicology in Cornell University Medical College, New York City. Fifth edition. Octavo, pp. 689. New York: William Wood & Company. 1902. [Price, $3.50 net.]

The first edition of this textbook for students was issued when Professor Witthaus was teaching chemistry at the University of Buffalo. It was used by his pupils then, and it has been their guide in the author's later classes at New York.

Changes have been made in this edition, notably the extension of the section in chemical physics and the condensation of that on mineral chemistry, but in which are now considered the study of the philosophy of chemistry and the broader principles of chemical science. The section on organic chemistry has been enlarged and partly rewritten, and the scattering references to subjects belonging to the domain of physiological chemistry in previous editions have been grouped in a single section, and treated in a more extended fashion. The section on laboratory technics has been omitted, as they belong to the laboratory handbook.

These changes cannot but make for improvement, an objective which this author is ever striving to reach, and they increase the value of the manual very materially. It is a book that students of chemistry will continue to find of great practical help, and the teacher will also use it as a guide to much of his work.

---

Transactions of the Medical Society of the State of New York. Ninety-sixth Annual Meeting held at Albany, January 28, 29 and 30, 1902. Frederic C. Curtis, M. D., Secretary. Published by the Society. 1902.

This volume is unusually replete with interesting material. It contains 62 papers on scientific medical subjects, besides all the other topics dealt with by standing and special committees, whose reports are printed in full. A semiannual meeting was held in New York during the year 1901, and the papers read at that meeting are included in this book.

The attendance at both annual and semiannual meetings was unusually large—363 at the latter and 470 at the former. The large attendance at the semiannual meeting was due no doubt in part to its novelty, and in other part to the fact that it was held in the City of New York at a season of the year when everybody who can do so enjoys a visit to the metropolis. It is doubtful, however, if it is wise for the society to indulge in this luxury of a semiannual meeting until it makes the regular annual session at Albany all it should be. The third day of that meeting is a stain upon the reputation of this great society. Something should be done to make it as interesting and as well attended as either of the other days.

This volume is beyond the average of state society transactions and ought to be read by every member.

A Treatise on Diseases of the Anus, Rectum and Pelvic Colon. By James P. Tuttle, A. M., M. D., Professor of Rectal Surgery in the New York Polyclinic Medical School and Hospital. Octavo, pp. 980. Illustrated. New York: D. Appleton & Co. 1902. [Price, cloth, $6.00; half leather, $6.50.]

The literature of the department of medicine upon which this book discourses with such signal ability, has been enriched very much of late not only in textbooks, but through valuable contributions to magazines.

In the present instance a large special clinic has furnished the material for a treatise not excelled in size or worth by any previous work. Passing over the first two chapters, one dealing with embryology, anatomy and physiology, and the other presenting malformations of the rectum and anus (both of which, however, are excellent and full of interest) we reach chapter three, which is allotted to examination and diagnosis. These topics form the foundation for the entire structure and have been treated with much elaboration in a chapter of 44 pages. It deserves to be carefully studied by undergraduate and general practitioner.

From this point onward the whole treatise is devoted to the consideration of diseases which affect the pelvic colon, rectum, and anus. In the descriptions Tuttle is clear, speaking generally, and his methods, including technic and treatment, are beyond captious criticism. If here and there are found points of difference between Tuttle and other men of experience they are for the most part, of minor consequence, and do not affect in particular the general statement that the book as a whole represents the present state of that branch of the science of medicine which, for the sake of convenience, we are accustomed to term proctology.

In the section relating to venereal diseases of the anus and rectum there is much information of great value from a clinical viewpoint. In dealing with chancroid of the anus, the author details the two theories of origin and regards the question of the presence of a specific virus, as still *sub judice*. It would be interesting to know which view of the subject Tuttle favors. We regard it as important that this doubtful condition in which the mass of the profession finds itself should be settled, and a man with such large clinical opportunities as Tuttle commands can do much towards determining it.

The chapter on hemorrhoids, consisting of 85 pages, is one of the most valuable in the book because it treats of a disease that is more often met than any other which afflicts this region.

It is particularly a disease upon which the general practitioner needs important light. He generally treats it with an "ointment" that is supposed to be the *summum bonum* of therapeutic resource in piles. In this chapter, which we wish might be published as a monograph and given general distribution, will be found very nearly the last word on the subject as at present understood.

The general make-up of the book is most satisfactory. It is a frank presentation of the author's experience and is a substantial addition to the literature of medicine. The illustrations deserve a word of praise being quite largely original in character and instructive in delineation. We bespeak a large audience for this painstaking author.

THE PHYSICIANS' VISITING LIST (LINDSAY AND BLAKISTON'S) FOR 1903. Philadelphia: P. Blakiston's Son & Company, 1012 Walnut Street. Sold by all booksellers and druggists. Price, $1.00 for 25 patients a week; $1.25 for 50 patients; $2.25 in two vols. for 100 patients.

The season of the visiting list is again upon us, and the first in the field, as it was originally the first published, is our old friend the Lindsay and Blakiston book. We confess that it has always been a favorite with us on account of its simplicity, compactness, and excellence in quality. There are no waste pages in it, while at the same time it contains all that is needful in such a pocket-book. Among the most useful portions of the preliminary material is the dose-table given in apothecary's and metric systems. The general style of the book is unchanged, though each year witnesses an improvement here and there, in order to keep it up to the requirements of the present, as indicated by experience. This is an age in which a visiting list is an essential part of a physician's equipment and should he choose this one he will have abundant reasons for satisfaction.

---

THE MEDICAL RECORD VISITING LIST OR PHYSICIANS' DIARY FOR 1903. New revised edition. New York: William Wood & Company.

This visiting list is one of the most popular published, having been issued successively for a long period of years, and apparently it has given general satisfaction. A complete revision of the reading matter in the front part of the list has been made this year. The table of average doses has been carefully revised and brought up to date, all the newer drugs of importance being included. A novelty now introduced for the first time into a visiting list is the obstetrical chart, facing page 4—a device that will be found useful for making quick estimates of the probable duration of pregnancy. In addition it contains all the usual material found in visiting lists, and arranged in a most convenient manner, the price of the book being from $1.25 to $4.00, according to size and style preferred. The ordinary book adapted to the needs of the average practitioner is arranged for 30 and 60 patients a week and sells at $1.25 and $1.50. The great demand for this diary makes it desirable for those contemplating its use to place their orders promptly, else there may be delay in delivery.

---

THE PHYSICIAN'S POCKET ACCOUNT BOOK, consisting of a manila-bound book of 208 pages and a leather case. By J. J. TAYLOR, M. D. Price, $1.00 complete. Subsequent books to fill the case, 40 cents each, or 3 for $1.00. Published by the Medical Council, Twelfth and Walnut Streets, Philadelphia.

A year ago, upon the first appearance of this unique and practical account book, we gave an unqualified approval of it, because we believed it offered the best condensed system of bookkeeping that had been devised for physicians. We are glad to have the opportunity to confirm our then expressed opinion after practical experience in its use.

The principal advantages of this account book are, that it is simple; that it is legal and will be admitted in court to prove indebtedness; that no posting is necessary, each entry being complete; that it can be carried in the pocket with convenience, hence may be referred to at any time—an important consideration in the country, where the physician is often asked for his bill at considerable distance from home. These and many other points which, for lack of space, we are unable even to enumerate, serve to make it a practical saver of time and money for those who use it. We are glad to be able to say this about the book for, as a rule, we have not been favorably impressed with special systems for physicians' accounts.

---

THE MEDICAL NEWS VISITING LIST FOR 1903. Weekly (dated for 30 patients); Monthly (undated, for 120 patients per month); Perpetual (undated for 30 patients weekly per year); and Perpetual (undated, for 60 patients weekly per year). The first three styles contain 32 pages of data and 160 pages of blanks. The 60-patient Perpetual consists of 256 pages of blanks. Each style in one wallet-shaped book, with pocket, pencil and rubber. Seal Grain Leather, $1.25. Thumb-Letter Index, 25 cents extra. Lea Brothers & Co., Publishers, Philadelphia and New York.

This useful visiting list is offered with its usual promptitude and presents an attractive appearance. Its arrangement is admirable and it contains all needful clinical data for daily or emergency use. In the descriptive heading will be found important facts that will enable a physician to choose the book best suited to his needs. The most useful part of every visiting list is the daily record of patients, and in this book the ruling into sections of five lines enables the eye readily to catch the information sought after entries have been made. Very little that is new can be said in regard to visiting lists in general or in particular, but we may add for the information of those who are selecting for the first time that the *Medical News* visiting list is issued in four styles, adapted to any system of records and any method of keeping professional accounts. It is printed on fine, tough paper, suitable for pen and pencil, and durably and handsomely bound in the size of a wallet for the pocket. When desired a thumb-letter index is furnished, which is an economiser of time.

